# Placement matching of alcohol-dependent patients based on a standardized intake assessment: rationale and design of a randomized controlled trial

**DOI:** 10.1186/s12888-014-0286-8

**Published:** 2014-10-14

**Authors:** Angela Buchholz, Anke Friedrichs, Michael Berner, Hans-Helmut König, Alexander Konnopka, Ludwig Kraus, Levente Kriston, Heinrich Küfner, Daniela Piontek, Fred Rist, Jeanette Röhrig

**Affiliations:** Department of Medical Psychology, University Medical Centre Hamburg-Eppendorf, Martinistraße 52, Hamburg, 20246 Germany; Department of Psychiatry and Psychotherapy, University Medical Centre, Hauptstraße 5, Freiburg, 79104 Germany; Department of Health Economics and Health Services Research, University Medical Centre Hamburg-Eppendorf, Martinistraße 52, Hamburg, 20246 Germany; IFT Institut für Therapieforschung, Parzivalstr. 25, Munich, 80804 Germany; Centre for Social Research on Alcohol and Drugs, Stockholm University, Stockholm, 106 91 Sweden; Department of Clinical Psychology and Psychotherapy, University of Münster, Fliednerstraße 21, Münster, 48149 Germany

**Keywords:** Placement-matching guidelines, MATE, Level of care, Alcohol dependence, Health-services research

## Abstract

**Background:**

Despite considerable research on substance-abuse placement matching, evidence is still inconclusive. The aims of this exploratory trial are to evaluate (a) the effects of following matching guidelines on health-care costs and heavy drinking, and (b) factors affecting the implementation of matching guidelines in the treatment of alcohol-dependent patients.

**Methods:**

A total of 286 alcohol-dependent patients entering one of four participating detoxification units and having no arrangements for further treatment will be recruited. During the first week of treatment, all patients will be administered Measurements in the Addictions for Triage and Evaluation (MATE), European Quality of Life-Five Dimensions health status questionnaire (EQ-5D), and the Client Socio-Demographic and Service Receipt Inventory—European Version (CSSRI-EU). Patients who are randomly allocated to the intervention group will receive feedback regarding their assessment results, including clear recommendations for subsequent treatment. Patients of the control group will receive treatment as usual and, if requested, global feedback regarding their assessment results, but no recommendations for subsequent treatment. At discharge, treatment outcome and referral decisions will be recorded. Six months after discharge, patients will be administered MATE-Outcome, EQ-5D, and CSSRI-EU during a telephone interview.

**Discussion:**

This trial will provide evidence on the effects and costs of using placement-matching guidelines based on a standardized assessment with structured feedback in the treatment of alcohol-dependent patients. A process evaluation will be conducted to facilitate better understanding of the relationship between the use of guidelines, outcomes, and potential mediating variables.

**Trial registration:**

German Clinical Trials Register DRKS00005035. Registered 03 June 2013.

## Background

Patient-treatment matching in substance abuse treatment (SAT) has been the subject of extensive research. The hope has been to improve treatment outcomes by allocating each patient to the *best-fitting* treatment option. Whereas attempts to match patient characteristics with treatment modalities have so far been disappointing [1,2], there is evidence individual patients’ needs can be successfully matched with different treatment services [3,4] and treatment intensities [5-7].

In order to implement patient-treatment matching in routine care, matching guidelines have been developed and evaluated in several countries. The guidelines usually define a set of criteria that can be used to determine an appropriate treatment or level of care (LOC) for each patient. In the United States, the Patient Placement Criteria of the American Society of Addiction Medicine are the most frequently used and evaluated placement criteria [4,8,9]. Recently, in the Netherlands matching guidelines were developed, evaluated, and implemented in the context of a nationwide reorganization of the SAT [10-12]. This approach was based on existing evidence on the efficacy of placement matching; it combines the concept of stepped care with the matching of treatment services according to patients’ needs [11,12]. Depending on their history of substance abuse treatment and their degree of impairment along the dimensions of *addiction severity*, *psychiatric impairment*, and *social instability*, patients can be assigned to brief outpatient treatment (LOC1), outpatient treatment (LOC2), day/residential treatment (LOC3), or inpatient or outpatient long-term care (LOC4; [11]). *Measurements in the Addictions for Triage and Evaluation* (MATE) [13] is the assessment instrument that was developed to implement these guidelines. The MATE is feasible for use in routine care, and it provides all of the information necessary to arrive at a recommendation of one of four LOCs according to the matching guidelines [14]. Studies on the feasibility and predictive validity of the Dutch guidelines have shown promising but inconclusive results. That is, the guidelines have been implemented successfully and have been shown to be feasible to use in routine care [11], but there was no effect on drinking outcome at a nine-month follow-up assessment. The authors, therefore, recently concluded that the matching guidelines need to be revised [15]. By now, necessary revisions are still being discussed in the Netherlands. It should be recognized, however, that various complexities are involved in using matching guidelines and various factors affect how effective using them is. This includes the structure of the particular SAT service in which they are used, the number of agents involved in the decision-making process (e.g. patients, therapists, treatment centres, funding agencies), and regional variations in the availability of treatment options. It is therefore appropriate to evaluate matching guidelines according to the guidance for the development and evaluation of complex interventions [16].

**Figure 1:**
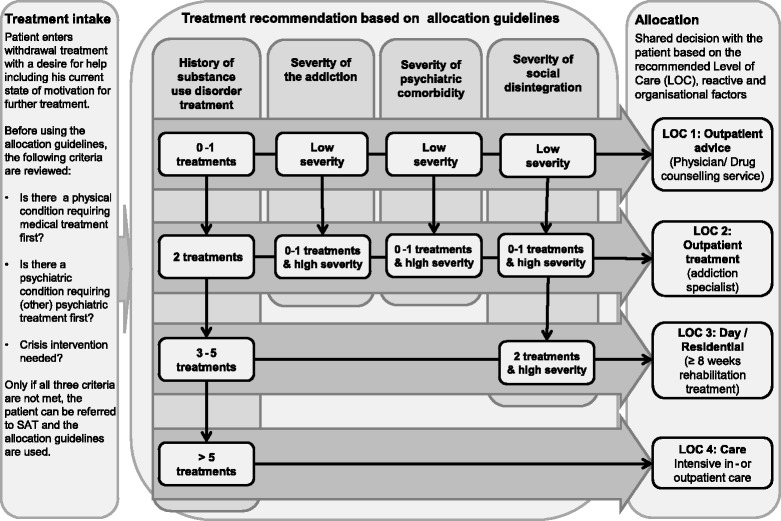
**Adapted allocation guidelines for referral decisions after detoxifications.**

The use of matching guidelines in the German SAT has also been the subject of discussion [17]. Recently, our study group conducted two pilot studies on the feasibility of using matching guidelines for patients with alcohol dependence following their participation in an inpatient withdrawal program. *Qualified withdrawal treatment* is a German-specific treatment program that includes, in addition to medically supervised detoxification, several therapeutic components aimed at enhancing patients’ motivation to remain abstinent, and referral for further treatment, if this is indicated [18]. Placement-matching decisions are often made during qualified withdrawal treatment. Our first pilot trial that included 54 alcohol-dependent patients in qualified withdrawal treatment indicated that both the setting and the procedures of the study would be feasible with minor adaptations [19]. In a second preparatory study using Delphi methodology, we asked experts in the German SAT to identify all relevant treatment options for patients with alcohol-use disorders in Germany and to organize them into four LOCs based on current evidence and discussions among the experts [20]. As a result, minor adaptations in the matching guidelines were made (see Figure 1).

## Methods

### Trial objectives and research questions

The objectives of this study are the evaluation of the matching approach that was previously adapted to include (a) using the MATE as the standardized intake assessment, (b) a treatment recommendation for each patient based on the MATE results, and (c) a feedback session with the patient. Following the recommendations developing and evaluating complex interventions [16], we are combining evaluation of the efficacy of the intervention with a process evaluation [21]. The hypotheses regarding the effects of the matching guidelines on reduction in alcohol consumption and health service costs are as follows:

H1: Patients in the intervention group (IG) will have fewer heavy-drinking days during the 30 days prior to the follow-up interview than those in the control group (CG).

H2: Patients in the IG will have lower health-service costs during the six months after the initial assessment than those in the CG.

The process evaluation will address the following research questions:

R1: How are trial outcomes related to variations in the extent and quality of the implementation of the intervention? We expect differences between study sites due to regional variations of the participating SAT.

R2: What are the factors that mediate the effects of the intervention received on treatment outcome? Mediating variables are expected to occur at the patient level (i.e. motivation, treatment preference, sociodemographic and clinical characteristics), the treatment level (i.e. recommendations that the therapists make, overall treatment effects), and the regional level (i.e. regional variations in the availability of treatment options, whether or not funding agencies approve the treatment). Previous studies regarding placement matching imply that there may be more variables mediating the effect of the intervention than captured in the actual matching algorithm. Due to a lack of evidence for major changes to the algorithm before starting the trial, it was decided to include those variables as possible mediators.

R3: Are there subgroups that differ in their response to the intervention? We expect that patients having specific treatment needs, e.g. pregnant women or patients with severe co-morbid psychiatric or physical disorders, might differ from other patients in their response to the intervention.

### Trial design and setting

The study is being conducted as a two-arm randomized controlled trial in four German detoxification wards offering inpatient withdrawal treatment for alcohol dependence, which lasts up to three weeks. All participating detoxification wards are located in psychiatric clinics and are specialized in withdrawal treatment for alcohol dependence. Patients with another primary or secondary psychiatric diagnosis are sometimes also referred to these wards. The assessment is being given during the first week of treatment, at treatment discharge, and six months after discharge. In the IG, an additional assessment will be given immediately after the intervention has been completed.

### Sample

The study will include patients with a primary diagnosis of alcohol dependence who are admitted to a qualified withdrawal program and sign an informed consent to take part in the study. Each patient’s therapist, who is a psychiatrist or a psychotherapist, will confirm the diagnosis or diagnoses at treatment entry. Exclusion criteria include being in treatment for reasons other than alcohol dependence, in crisis and needing crisis intervention, severely cognitively impaired, psychotic, illiterate, or having insufficient German language skills. Because the aim of the study is to evaluate referral decisions, an additional exclusion criterion is already having finalized plans for referral for subsequent treatment.

### Measurement

The MATE [13] is a semi-structured interview that is based on the World Health Organisation’s biopsychosocial model of health [22]. It includes 10 modules assessing alcohol and drug use during the past 30 days and the person’s lifetime, substance abuse treatment history, psychiatric and physical co-morbidity and symptom severity, diagnosis of substance -use disorder and its consequences for the person’s everyday life, need for care, and social stability. The MATE yields 20 sum scores, which again can be summarized into four dichotomized so called dimension scores: Addiction severity, severity of psychiatric co-morbidity, severity of social disintegration, and history of treatment for a substance-use disorder. With the use of these four dimensions, a recommendation for referral to one of four LOCs can be made [13]. A computer-assisted version of the MATE is available; administering it takes approximately 45 minutes to complete. The MATE-scores, including the recommendation of one of the four LOCs (LOC 1: Outpatient advice; LOC 2: Outpatient treatment; LOC 3: Day/Residential treatment; LOC 4: Care), are calculated automatically. The MATE has been shown to have acceptable psychometric properties, and it was found to be feasible for use in routine care and in research settings in both the Netherlands and Germany [14,23].

The MATE-Outcomes is an abbreviated version of the MATE, which was developed as a follow-up assessment for treatment evaluation purposes.

The Client Sociodemographic and Service Receipt Inventory (CSSRI-EU) is a standardized Europe-wide validated instrument to evaluate health-service utilization and medication use for the domain of mental-health care [24]. Using the data on health service utilization, health care costs can be estimated in a second step via monetary valuation with unit costs. The CSSRI-EU can be conducted as an interview; it takes approximately 20 minutes to complete. The validity of the German version of the instrument [25] and cost implications based on the CSSRI-EU have been demonstrated [26].

The European Quality of Life-Five Dimensions health status questionnaire (EQ-5D) is a short generic quality-of-life assessment [27] comprising EQ-5D five items: Mobility, self-care, usual activities, pain/discomfort, and anxiety/depression [28]. A five-digit number is derived that shows the person’s self-reported state of health. A visual analogue scale (EQ VAS), ranging from (0; worst imaginable health state) to (100; best imaginable health state) is also used to summarize the patient’s overall health state. The acceptance and validity of the EQ-5D for alcohol-dependent patients has been reported [29].

### Process evaluation measures used during treatment

A structured documentation form, which we developed, will be used to record all recommendations regarding further treatment that are given to the patient during withdrawal. For the patients in the IG, this includes (a) the LOC that is recommended based on the MATE, (b) therapists’ dissenting recommendations, if they occur, and reasons for the disagreement, (c) results from the feedback session, and (d) the final referral decision that was made at discharge. For patients of the CG, referral decisions are documented at discharge, using the same documentation form that is used for patients in the IG. In addition, research assistants complete a documentation form, which includes the number of patients who are eligible to participate in the study, the number of patients who agreed to participate, and for patients of the IG the number of referral decisions that are concordant with the recommendation that was derived from the MATE.

Patients are also asked to complete a questionnaire, which includes questions about alcohol use and psychosocial problems [30], the patients’ preferences regarding further treatment, and the Control Preference Scale (CPS) [31]. With the CPS, role preferences of patients regarding medical decision making, i.e. a passive, shared or active role, are assessed.

### Primary outcomes

The primary outcome measures will be alcohol consumption and health-care costs; they will be assessed six months after discharge from the current withdrawal treatment. Regarding alcohol consumption, per cent days of heavy drinking in the last 30 days (PDHD) was chosen as the primary outcome measure [32]. It is derived from the MATE-Outcomes*.* Health-care costs are estimated based on data on health-service utilization as assessed with the CSSRI-EU. The estimate includes a monetary valuation with unit costs.

Outcomes for the process evaluation include quantitative and qualitative data. In addition to the assessment instruments and documentation forms used during the trial, after the data have been collected, research assistants and heads of the participating treatment wards will be invited to attend a focus group to discuss potential benefits from and barriers for an implementation of the matching guidelines.

### Intervention

After the MATE interview with patients in the IG, the research assistant will review with the patient’s therapist or social worker, or both, the LOC recommendation based on the MATE results. In a subsequent feedback-session, the research assistant will explain the recommendation and will strive to reach a consensual decision with the patient regarding further treatment. In case a therapist has a diverging recommendation, this revised recommendation will be explained to the patient. At the end of the feedback session, the decision that the patient and research assistant have made will be documented, and the therapeutic staff will be informed about the decision.

Patients in the CG will not have an additional feedback session regarding their further treatment options and are treated as usual. These patients will receive global feedback regarding their assessment results, without a recommendation for a LOC, if they request it.

### Procedure

Immediately after admission to the withdrawal unit, patients will be invited to take part in the study. A research assistant will inform patients about the study procedure, and patients who wish to participate will be asked to sign an informed consent. Patients who have done so will be asked to complete a questionnaire prior to their first assessment. This assessment will be scheduled when a patient’s withdrawal symptoms have decreased to a minimal level as judged by the medical staff. During the assessment interview, a research assistant will administer the MATE, CSSRI-EU, and EQ-5D. Finally, the research assistant will be informed by the computer-assisted MATE, whether the patient has been randomly assigned to either the IG or the CG. For patients in the IG, the research assistant will schedule the feedback session.

**Figure 2:**
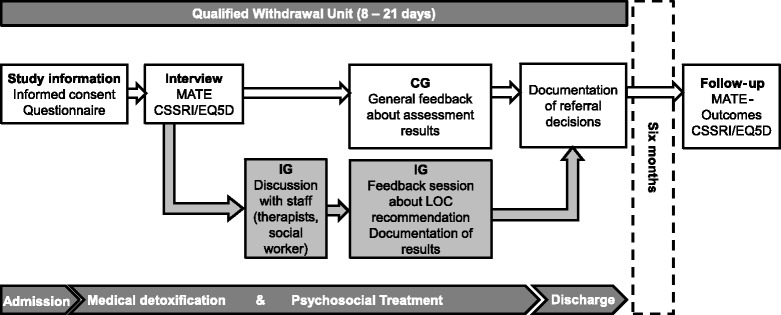
**Flowchart of patient progress through the qualified withdrawal unit and study procedure; MATE = Measurements in the Addictions for Triage and Evaluation; CSSRI = Client Sociodemographic and Service Receipt Inventory; EQ5D = EQ-5D Health questionnaire; IG = Intervention group; CG = Control group.**

At the end of the withdrawal treatment, all decisions and arrangements regarding subsequent treatment will be documented for patients in both the IG and the CG. Six months after discharge from the withdrawal treatment, patients are contacted for a telephone follow-up interview, which will include the MATE-Outcomes, CSSRI-EU, and EQ-5D. Patients who complete the entire procedure including the follow-up assessment will receive payment of 30 euros. The study procedure is diagrammed in Figure 2.

### Randomization

Randomization occurs by means of computerized blocked randomization with varying block sizes that are stratified by trial site. Research assistants are blinded until the first assessment has been completed.

### Sample-size calculation

**Figure 3:**
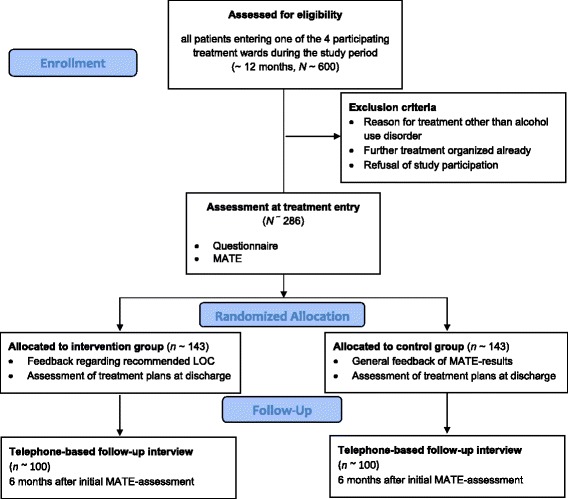
**Approximated patient flow of MATE-LOC.**

Due to the exploratory nature of the trial, the sample-size calculation was based on considerations regarding the process evaluation rather than the primary outcome measures. The concordance between MATE recommendations and actual referrals to the recommended LOC was considered to be an important mediator between the intervention and the outcome measures. Because classification into concordant (matched) and discordant (mismatched) decisions yields a cross-classification in addition to the LOC groups and study arms, it was important to ensure that in both study arms all of the cells include at least 10 analyzable cases at the six-month follow-up. Based on prior studies [11,19], we expected an imbalance between the four LOCs (approximate ratio: 1:3:5:1). Based on these assumptions, 100 analyzable cases would be needed in each arm at follow-up. Assuming a drop-out rate of 30 % between randomization and follow-up, 143 patients should initially be included in each study arm (or 286 patients in total). This sample size would ensure that subgroup analyses can be conducted, even if attrition rates are higher than expected or they differ among study arms or LOCs. Figure 3 shows the expected flow of patients.

### Statistical analyses

All of the primary analyses will be conducted according to the intention-to-treat principle (ITT). There is a risk that not every patient in the IG will have a feedback session during his or her withdrawal treatment. This could result from time constraints for the therapists, unscheduled dropouts from treatment, or premature referrals for subsequent treatment. Therefore, additional per-protocol analyses will be conducted that include only those patients who actually participated in the intervention. In order to evaluate the robustness of the results, missing data will be addressed by using at least two procedures (e.g. complete case analyses, imputation by expectation-maximization, multiple imputation by chained equations), depending on the outcome and the patterns in the missing data. Differences between the two treatment arms regarding the primary outcomes will be analyzed using generalized linear models with gamma distribution and log-link function controlling for confounders (e.g. baseline costs and PDHD, age, gender, co-morbidity, EQ-5D scores). To account for correlated patient data caused by the multicenter structure, random-effect models will be applied.

Subgroup analyses will include effects of the classification as matched vs. mismatched decisions for patients in both the IG and the CG. That is, patients who received treatment according to MATE guidelines will be designated matched*,* and those who did not will be designated as mismatched. The effect of matching MATE recommendations with treatment received on the primary outcome measures will be analyzed using multiple linear regressions. Therapists’ documented reasons for deciding to deviate from MATE-LOC recommendations will also be analyzed in order to decide whether the decision algorithm should be modified. Additional exploratory analyses will be conducted to determine the relevance that patients’ demographic and clinical characteristics, their motivation for treatment, and their treatment preferences has for patient-treatment matching.

### Data monitoring and quality assurance

Research assistants will receive two days of training in the study procedures, data monitoring, how to administer the assessments, and how to conduct the feedback sessions. All assessment interviews and feedback sessions will be audiotaped. The coordinator of the study will supervise every fifth interview and feedback session to ensure that the research assistants adhere to the intended protocol. Data monitoring will be under the auspices of the Institut fuer Therapieforschung (IFT). Each study center will send all of the assessments conducted during the withdrawal treatment to the IFT. In turn, a research assistant at the IFT will continuously review the data sets and will report missing or incomplete data to the study center. If possible, research assistants complete missing data and then report back to the IFT.

### Confidentiality and ethical approval

Both the ethics committee of the local medical association in Hamburg, the *Ethik-Kommission der Ärztekammer Hamburg* (Reference Number PV4325) and the ethics committee at each of the participating sites have approved the study protocol, including all of the patient information that will be collected, and the informed consent documents that patients will be asked to sign. Ethical approval was granted in accordance with the principles of the Declaration of Helsinki [33]. Patients’ participation in the study will be voluntary, and confidentiality will be assured. Because participants’ names and telephone numbers will be needed in order to contact them for the follow-up interview, the data cannot be stored anonymously until after the follow-up assessments have been completed. Prior to this, pseudonyms will be used, and personal data will be stored separately. When the follow-up assessments have been completed, the personal data will be deleted.

## Discussion

Based on a randomized controlled design, the current study will empirically evaluate a matching procedure that includes a standardized assessment, a recommendation derived from the assessment results, and a feedback session. Thus, the study will advance the work of Dutch colleagues who designed the MATE and the MATE matching algorithm and conducted large-scale naturalistic trials involving the entire Dutch SAT [10-12,14,15]. In the current study, we have the opportunity to (a) use a randomized controlled design, and to (b) evaluate the matching approach in a different health-care system. There are, however, several potential threats to consider in conduct of the study and in analyzing and interpreting the results.

First, the proposed intervention combines several steps: Evaluation of each patient’s eligibility for the matching process; a LOC recommendation based on the patient’s MATE, results; and the therapist’s possible modified recommendation, which would be explained to the patient during a feedback session. At this time, we cannot estimate how often the therapists will deviate from the original LOC recommendation. Another important factor that can affect referral decisions is whether or not insurance agencies (i.e. health insurance, pension funds, or social welfare) approve and finance the treatment that is recommended. As part of our follow-up assessment, we will record whether patients applied for a treatment that was not approved. We can, therefore, estimate the frequency with which applications are not approved and take this into consideration when making the LOC recommendations in the process-evaluation phase of the trial.

In this trial, we are focusing on the time period immediately after the withdrawal treatment and up to six months after treatment. This time period might be too short to reliably estimate differences between the groups in health-care costs and alcohol use. This follow-up interval, however, was chosen for practical reasons. Moreover, it will allow us to closely monitor actual referrals following withdrawal and determine how they are related to the LOC that was recommended. If this approach succeeds in allocating patients completing the withdrawal treatment to appropriate subsequent treatment, a larger trial will be needed to derive a more valid and reliable estimation of the effects.

The treatment as usual during qualified withdrawal treatment may have an impact on the ability of the study design to detect group differences. Since one part of the treatment includes discussions regarding further treatment, differences between IG and CG may be decreased. However, the choice of the treatment setting for this study has been made for several reasons and has been piloted in a previous study (19). In contrast to the Dutch studies, this study will include only one part of the German SAT, i.e. qualified withdrawal treatment for patients with alcohol dependence. Matching processes do, however, occur in several other parts of the German SAT (e. g. drug counselling services) and in primary care. Additionally, systematic integration of decisions to refer patients with co-morbid disorders other than substance use to psychiatric treatment or social care might need to be made. By including a process evaluation, we expect to obtain initial answers that can serve as the basis for further discussion. How to evaluate the generalizability of this approach for other parts of the German SAT will be a major issue in the discussion and interpretation of the results. In order to achieve a broad and successful implementation of the matching guidelines, much political and scientific discussion will be needed, and it will be necessary to integrate them with existing national treatment guidelines.

### Trial registration and status

This study is registered with the German Clinical Trials Register as Trial DRKS00005035. Date of registration: 03/06/2013. Recruitment of participants started in June 2013, and testing participants, including the follow-up assessments, is expected to be completed in December 2014. This is the first draft of the study protocol, there have been no amendments or changes made in the trial design. Date of first submission: 28.04.2014. Date of second submission: 3.09.2014.

## Abbreviations

CG: Control group
CPS: Control Preference Scale
CSSRI-EU: Client Socio-Demographic and Service Receipt Inventory—European Version
DRKS: German Clinical Trials Register
EQ-5D: European Quality of Life-Five Dimensions health status questionnaire
EQ VAS: European Quality of Life-visual analogue scale
IFT: Institut fuer Therapieforschung
IG: Intervention group
ITT: Intention- to-treat
LOC: Level of care
MATE: Measurements in the Addictions for Triage and Evaluation
PDHD: Percent days of heavy drinking
SAT: Substance abuse treatment
